# Effect of soy on faecal dry matter content and excretion of *Brachyspira hyodysenteriae* in pigs

**DOI:** 10.1136/vetreco-2015-000159

**Published:** 2016-05-02

**Authors:** Alexander Grahofer, Gudrun Overesch, Heiko Nathues, Friederike Zeeh

**Affiliations:** 1 Department of Clinical Veterinary Medicine, Vetsuisse Faculty, Clinic for Swine, University of Bern, Bern, Switzerland; 2 Department of Infectious Diseases and Pathobiology, Vetsuisse Faculty, Institute of Veterinary Bacteriology, University of Bern, Bern, Switzerland

**Keywords:** Bacterial diseases, Nutrition, Diagnostics, Enteric disease, Pigs, Spirochaetes

## Abstract

The aim of this study was to investigate the effect of a soy diet on the excretion of *Brachyspira hyodysenteriae* in five farms with subclinically infected pigs. The effects on general health, faecal consistency and dry matter were analysed. In total, 200 pigs of different ages (group 1 <100 days of age (n=120) and group 2 ≥100 days (n=80)) were randomly assigned to the control (C) and the treatment (T) groups. Group C received the farm's standard diet. In group T half of the daily feed ration was replaced by pure soy on two consecutive days. Faecal scores were used to determine faecal consistency and a microwave method to assess faecal dry matter content (FDMC). In age group 1, soy feeding resulted in a statistically significant decrease of the FDMC of 2.5 per cent compared with group C and in age group 2 in a significant increase of 2.2 per cent compared with group C at day 2. Overall seven (T: 5, C: 2) out of 597 faecal samples tested positive for *B hyodysenteriae* by PCR.

In conclusion, a high soy diet applied over two days influenced the faecal consistency and the FDMC in growers, finishers and sows under field conditions. Further investigations with more sensitive diagnostic methods are needed to prove a potential influence of a high soy diet on the detection rate of *B hyodysenteriae* in subclinically infected herds.

## Introduction

*Brachyspira hyodysenteriae*, a Gram-negative anaerobic intestinal spirochaete, is the causative agent of swine dysentery (SD). SD is a severe mucohaemorrhagic enteric disease, widespread in most countries and has a large impact on pig production due to tremendous financial losses (Alvarez-Ordóñez and others 2013, Wilberts and others 2015). *B hyodysenteriae* is transmitted horizontally by ingesting contaminated faeces. Clinical signs of diarrhoea with mucus and blood in the faeces are usually observed in growers and finisher pigs, but subclinical disease can also occur (Alvarez-Ordóñez and others 2013). Pigs can carry *B hyodysenteriae* for up to 70 days without any clinical signs and may be a source of infection for other pigs or herds.

Therefore, an accurate diagnostic tool is inevitably needed for an efficient therapeutic approach and to support monitoring programmes in the field. Traditional diagnostics are culture-based, often performed on trypticase soy agar, to detect the pathogen; biochemical tests are performed for identification (Wilberts and others 2015). This procedure is very laborious, time-consuming and expensive, as *B hyodysenteriae* is fastidious and grows slowly (Råsbäck and others 2006, Song and Hampson 2009). Influenced by these constraints, several PCR assays have been developed during recent years (La and others 2003, Råsbäck and others 2006, Nathues and others 2007, Song and Hampson 2009). Although it is highly sensitive, PCR can fail in cases of intermittent shedding (Jacobson and others 2004a) or in cases where only small amounts (approximately 10^2^–10^3^ colony-forming units per gram faeces) of *Brachyspira* species are present in the sample (Corona-Barrera and others 2004). A procedure leading to constant or elevated shedding of *B hyodysenteriae* in porcine faeces would be of great advantage generally and in cases of subclinical SD in particular. Feed has been identified as a risk factor for infecting pigs with *B hyodysenteriae* (Leser and others 2000, Durmic and others 2002, Jacobson and others 2004b)*.* Soybean meal is a common source of protein for pigs and is used in different proportions in the various age groups. . The major components of soy are more than 45 per cent raw protein, six per cent oligosaccharides and four per cent crude fibre (Baker and others 2011). Unbalanced feed and soy portions might well have an impact on pig health (Montagne and others 2003). Consequently, in experimental infection models for *B hyodysenteriae* a provocative feeding regimen with soy altered the microbial flora and encouraged the development of the disease (Durmic and others 2000, Jacobson and others 2004b).

However, to the authors’ knowledge, no study has focused on the potential effect of a high soy diet as a diagnostic approach in pig herds.

The gut health situation on a farm can be clinically assessed by evaluating faecal consistency in pig herds (Pedersen and others 2012). A more objective method is to measure the faecal dry matter content (FDMC) with a recently described microwave procedure (Pedersen and others 2011).

It was hypothesised that a high soy diet applied over two days might increase the shedding of *B hyodysenteriae* and therefore the detection rate of *B hyodysenteriae* in infected pigs*,* which would provide a convenient diagnosis in the field. PCR was chosen as a diagnostic tool to obtain a rapid result in a cost-effective way. Determining the faecal parameters should demonstrate the general effect of the soy diet on the pig’s gut.

The aims of the present study were to investigate the effect of a high soy diet on the general health of pigs, on the faecal consistency, on dry matter content and finally on the faecal excretion of *B hyodysenteriae* in pigs of different ages.

## Materials and method

An experimental field study was conducted in four grower-fattener herds and one breeding herd between April 2014 and January 2015 in Switzerland. The animal experiments in this study were approved by the Cantonal Veterinary Office Bern (Licence No. BE83/14).

The five farms were positive for *B hyodysenteriae*, which had been demonstrated between three and ten months before by culture (Dünser and others 1997) and PCR (La and others 2003), and they had neither undertaken any measures nor treated the affected animals to eradicate *B hyodysenteriae*.

The study comprised a total of 200 crossbreed pigs of different ages (40 growers of about 60 days of age, 40 growers of about 90 days of age, 40 growers of about 100 days of age, 40 finisher pigs of about 140 days of age and 40 sows between 246 days and 1928 days of age). The sample size was calculated based on the estimated differences between the FDMCs before and after soy feeding using the free software WinEPi (http://www.winepi.net). Differences had been determined in a preliminary study (Zeeh and others 2015). The number of 20 pigs per group would have been sufficient to detect an increase; that is, difference in prevalence of Brachyspira of 30 per cent (0 v. 30) accepting 95 per cent confidence and 80 per cent power. Exclusion criteria were feeding whey in the age group of interest, because of high potential variation in whey compositions (Johansen and others 2002), deworming during the trial and the use of *B hyodysenteriae* effective antimicrobials in the previous six months until the study. In addition, sows up to three weeks after insemination, up to three weeks before birth and during lactation were excluded.

**FIG 1:**
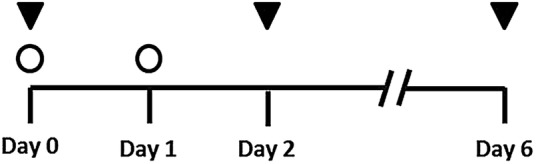
Time schedule of sample collection and treatment of 200 pigs in five herds. Filled triangles represent sampling days and unfilled circles represent treatment with a high soy diet in the treatment group (n=100 pigs)

Per herd, 40 pigs were selected for the study. In the breeding herd, sows were randomly selected according to parity, day of gestation and age. In the grower-finisher herds, pigs were stratified according to their age, depending on the present age groups. In a particular stratum, the 40 pigs were then randomly allocated by drawing lots either to the control group (20 pigs; group C) or to the treatment group (20 pigs; group T). The first day of the study was considered as D0. Group C pigs received the farm's commercial standard diet. In group T, half of the daily feed ration on D0 and day 1 was replaced by pure soy extraction grist (Fig 1). The time period of feeding soy had been evaluated in a preliminary study (Zeeh and others 2015), where the largest effect of soy on the faecal structure had been detected after two days of soy. The soy used in the preliminary and present study originated from Brazil, was not genetically modified, and had 48 per cent raw protein and 3.5 per cent crude fibre. All group T pigs received soy from the same batch of production. A clinical examination with focus on general health, feed intake and rectal body temperature was performed in all pigs included in the study on D0, day 2 (D2) and day 6 (D6) by the principle investigator (Fig 1). Rectal faecal samples and rectal swabs of each of the pigs were taken on the same days. For each pig, the examiner used a new pair of disposable gloves and rectal swabs were collected before measuring the rectal body temperature to avoid any contamination. The swab (Transwab, Medical Wire & Equipment, Wiltshire, England) was inserted into the rectum and twisted there for at least five seconds. The faecal swabs were slewed in an individual reaction tube containing 1 ml chaotrophic SV lysis buffer (4M guanidine thiocyanate, 0.01M Tris–HCl, 1 per cent b-mercaptoethanol) for at least 10 seconds. Rectal faecal samples (approximately 30 g) from every pig were taken and the faecal consistency was determined. A previously described faecal consistency scale with four descriptive categories (Pedersen and Toft 2011) had been modified by adding a fifth category. The five consistency scores were 1=firm and shaped, 2=soft and shaped, 3=loose, 4=watery and 5=watery with blood and/or mucus. The faecal samples were placed in individual, closed containers for further analysis in the laboratory.

The SV lysis buffer samples for detecting *B hyodysenteriae* genome fragments by direct TaqMan-PCR assay were transported at room temperature to the laboratory. Analyses were performed at the Institute of Veterinary Bacteriology at the Vetsuisse Faculty, University of Bern, as previously described (Song and Hampson 2009, Bürki and others 2011). Faecal samples were transported at room temperature and stored at 4°C for two to five days until further analysis. The FDMC was determined in triplicates with a modified microwave procedure (Pedersen and others 2011). A microwave oven (LIFETEC, Medion AG, Germany) with six different power levels and a maximum effect of 700 W was used. A glass Petri dish was weighed using a digital pocked scale with 0.01 g resolution (W1). Subsequently between 1.5 g and 2 g of faeces were weighed and the weight of the loaded Petri dish (W2) was recorded. Up to 24 subsamples were placed in the microwave and initially dried at 119 W for 30 minutes, followed by 10 minutes at 385 W. The individual weights were determined before all samples were reheated at the same power level for five minutes. Subsamples were weighed and reheated until two consecutive heating and weighing cycles resulted in the same weight (±0.01 g; W3). FDMC (per cent) was calculated using the equation ‘FDMC=(((W3−W1)/W2)×100)’.

Data were collected using structured and standardised data collection forms. All data from the five herds as well as results from laboratory testing were entered into a spreadsheet program (Microsoft Office Excel 2010). Continuous variables were tested for normality and homogeneity of variance. If these assumptions were met, then the two groups were evaluated using a Multivariate Analysis of Variance (MANOVA) model. The MANOVA model was used to show the effect of a high soy diet on faecal consistency, on FDMC and on the detection rate of *B hyodysenteriae* by comparing the basic values on D0 of group C and group T to the respective two post-treatment values (D2, D6). The Mann–Whitney U test was used to determine the differences of FDMC between the group C and the group T. Spearman's rank correlation coefficients between faecal consistency and FDMC in both age groups were derived. Data was analysed using NCSS V.9 (http://www.NCSS.com) and JMPPro V.10 (SAS Institute, Cary, North Carolina, USA), considering statistical significance when P<0.05.

## Results

In each of the five herds, 40 pigs were included in the study resulting in a total number of 200. All the five herds were managed with a continuous flow system. The median herd size of the four fattening herds was 275 animals (range 160–850). The total number of sows in the breeding herd was 148. The median age of all animals was 98 days (range 61–1928). The median parity of the sows was 4.5 (lower median=4, upper median=5; range 1–12) and the median gestation length at the time of enrolment was 60 days (range 26–84). The parity and the gestation length did not significantly differ between group C and group T. The standard feed ration on the sow farm contained 15 per cent raw protein and 8 per cent crude fibre. The fatteners received a standard diet with the median raw protein of 17.3 per cent (range 15.0–19.9) and the median crude fibre of 4.15 per cent (range 4.0–4.2). For further analysis pigs with an age <100 days (age group 1) and ≥100 days (age group 2) were analysed separately, because of known differences in digestive utilisation of feed ingredients depending on age (Le Goff and Noblet 2015). On D0, 200 pigs were included in the clinical examination and PCR analyses. On D2, 199 pigs and PCR samples were examined; one grower pig from group C was excluded from all succeeding examinations due to a prolapsed rectum. On D6, 198 pigs were included in the clinical examination and PCR analyses; one grower pig from group C was excluded due to sudden death. Lack of faecal samples or insufficient amount of faecal material was the reasons for various missing values in the data set.

### Clinical examination

None of the 198 pigs showed signs of reduced general condition or specific signs of SD throughout the entire study period. All pigs in both treatment groups consumed the diet. Rectal body temperature did not differ between the experimental groups on all three days of examination. The median rectal body temperature was 39.2°C (range 36.5°C–41.0°C) on D0 (n=200); 39.1°C (37.0°C–40.8°C) on D2 (n=199) and 39.1°C (37.2°C–40.7°C) on D6 (n= 198).

### Faecal consistency

The median faecal consistency in group C was 1.5 (range 1–4) on D0 (n=97), 1.5 (1–4) on D2 (n=98) and 1.5 (1–4) on D6 (n=96). The median faecal consistency in group T was 2 (1–4) on D0 (n=98), 2 (1–4) on D2 (n=95) and 2 (1–4) on D6 (n=99). When considering the two age groups, the median faecal consistency values in age group 1—treatment group C were 1.5 (1–4) on D0 (n=57), 1.5 (1–4) on D2 (n=58) and 2 (1–4) on D6 (n=57). The values in age group 1—treatment group T were 2 (1–4) on D0 (n=58), 2.5 (1–4) on D2 (n= 56) and 2 (1–4) on D6 (n=59). The faecal consistency values in age group 2—treatment group C were 1 (1–4) on D0 (n=40), 1.25 (1–2) on D2 (n=40) and 1 (1–3) on D6 (n=38); the values in age group 2—treatment group T were 1.5 (1–4) on D0 (n=40), 1 (1–3) on D2 (n=39) and 1 (1–3) on D6 (n=40).

### Faecal dry matter content

In 98.7 per cent of the cases, one heating cycle was sufficient to obtain a constant weight. On two occasions, the samples were burned or boiled. The analyses of these two samples were repeated.

The median FDMC in samples of group C was 22.65 per cent (range 7.00–29.49 per cent) on D0 (n=95), 22.48 per cent (6.09–29.3 per cent) on D2 (n=98) and 22.55 per cent (4.55–29.58 per cent) on D6 (n=94). The median FDMC in group T was 22.44 per cent (6.43–31.44 per cent) on D0 (n=97), 22.16 per cent (7.91–38.44 per cent) on D2 (n=95) and 22.47 per cent (8.12–34.37 per cent) on D6 (n=97). More details are presented in Table 1.

**TABLE 1:** Median faecal dry matter content of pigs on study day 0 (D0), day 2 (D2) and day 6 (D6) in the treatment group (group T) that received a high soy diet at study days 0+1 and the control group (group C) without soy supplementation

	Median faecal dry matter content (%) (range); number of tested animals (n)		
Groups	DO	D2	D6
Group (<100 days)	20.95 (6.43–29.49);* n=112	20.21 (6.09–29.3); n=114	20.85 (4.55–28.5); n=115
Group (≥100 days)	23.45 (11.06–31.44);* n=80	24.79 (17.55–38.44); n=79	24.58 (17.94–34.37); n=76
Group C (all pigs)	22.65 (7.00–29.49); n= 95	22.48 (6.09–29.3); n= 98	22.55 (4.55–29.58); n=94
Group T (all pigs)	22.44 (6.43–31.44); n=97	22.16 (7.91–38.44); n=95	22.47 (8.12–34.37); n=97
Group C (<100 days)	21.97 (7.00–29.49); n=55	22.18 (6.09–29.3); n=58	21.47 (4.55–28.16); n=57
Group T (<100 days)	21.89† (6.43–25.38); n=57	19.41† (7.91–29.13); n=56	21.31 (8.12–28.5); n=58
Group C (≥100 days)	23.25 (13.57–29.39); n=40	23.69 (17.55–28.73); n=40	23.78 (17.94–29.58); n=37
Group T (≥100 days)	23.55† (11.06–31.44); n=40	25.74† (18.79–38.44); n=39	24.37 (20.17–34.37); n=39

Both groups are also presented depending on the age groups (age group 1<100 days and age group 2≥100 days)

*Values of age group 1 (<100 days) and age group 2 (≥100 days) differ significantly (P<0.01)

†Values from D0 compared with D2 in age groups 1 and 2 differ significantly (P<0.01)

**FIG 2:**
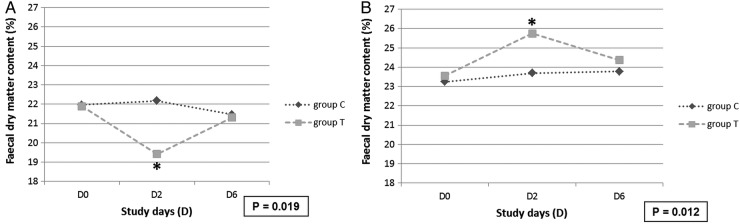
Average faecal dry matter content from pigs on study day 0 (D0), day 2 (D2) and day 6 (D6) in the treatment group (group T) that received a high soy diet at D0 and study day 1 and in the control group (group C) without soy supplementation. In (A) values of 105 pigs with an age <100 days and in (B) values of 76 pigs with an age ≥100 days are presented. A significant difference of the faecal dry matter content between the treatment group and the control group was detected for both age groups on D2

Baseline values of the FDMC, determined on D0, did not differ between the groups T and C. A statistically significant difference between age group 1 and age group 2 on baseline values of FDMC was detected (P<0.001). Faecal consistency score was negatively correlated to the faecal dry matter in both study groups (Table 2). Changes of the FDMC from the pigs from study day D0 to days D2 and D6 in the group T and group C and in the two age groups are shown in Fig 2.

**TABLE 2:** Linear correlation coefficient analysis of faecal dry matter content (FDMC) and faecal consistency (FC) from group T (with soy supplementation day 0 and day 1) and group C (without soy supplementation) (both groups n=576 samples) on the sampling day 0 (D0), day 2 (D2) and day 6 (D6)

Correlation between FDMC and FC on sampling days	Rho (ρ)	95% CI	P value
FDMC-FC D0	−0.8114	30.43 to 33.52	<0.0001
FDMC-FC D2	−0.8653	31.68 to 34.69	<0.0001
FDMC-FC D6	−0.8009	29.32 to 32.16	<0.0001

### Detection of *B hyodysenteriae*

PCR analyses were performed in 597 faecal swab samples.

In one finisher herd three samples from three different pigs (D0: 1 sample (group T); 1 sample (group C)); D6: 1 sample (group T) and two consecutive samples from one pig (D0; D2: (group T)) tested positive, which resulted in an average detection rate of *B hyodysenteriae* of 4.2 per cent at herd level. In one grower herd, two samples from two pigs (D6: 1 sample (group T); 1 sample (group C)) tested positive, which resulted in an average detection rate of 1.7 per cent. The overall detection rate on the three days of testing (D0, D2 and D6) varied from 0.5 per cent to 1.5 per cent. On all days of sampling, no statistically significant differences between experimental groups were detected.

## Discussion

Many studies investigating potential risk factors for *B hyodysenteriae* infection in pigs were predominantly focused on nutrition (Stege and others 2001, Wilberts and others 2014). In different experimental infection models for *B hyodysenteriae,* a provocative soy-feeding regimen enhanced the development of the disease. As subclinically infected pigs are considered to be important initiators for spreading the disease within and between herds (Alvarez-Ordóñez and others 2013), the present study was designed to investigate the effect of a high soy diet on the general health, changes of the faecal consistency and of dry matter content and on excretion of *B hyodysenteriae* in pigs of different ages. The potential influence on the detection rate of the pathogen was analysed by PCR testing.

Soy can induce nutritive diarrhoea in pigs (Neef and others 1994, Makinde and others 1996). Especially feeding a high soy diet in growers can cause changes in FDMC, due to non-digestible oligosaccharides like raffinose, stachyose and verbascose, which can decrease interaction with digestive enzymes in the intestine (Smiricky and others 2002) and increase the osmotic pressure in the colon, resulting in diarrhoea (Makinde and others 1996). In addition, a hypersensitivity reaction to soy protein can also cause malabsorption in the colon (Engle 1994) and changes in faecal dry matter. High soy diets might alter the intestinal milieu and produce favourable conditions for *B hyodysenteriae*, allowing the pathogen to multiply (Jacobson and others 2004b). In the present study, after two days of high soy diet, a significant decrease of FDMC could be detected in the age group 1 (pigs of 60–100 days of age). Interestingly, on D2, pigs of ≥100 days of age showed a significant increase in FDMC of 2.19 per cent points from 23.55 per cent to 25.74 per cent after feeding the soy. Sows have a better total digestibility (Bach Knudsen and others 2012, Le Goff and Noblet 2015) and greater number of microbes regardless of microbial adaption (Varel 1987) compared with growing pigs resulting in a higher water absorption in the large intestine. This might be one of the potential causes for the higher FDMC in age group 2 observed in the present study.

The modified microwave method was easy and fast for determining FDMC. A negative correlation between faecal consistency score and FDMC could be observed in the present study. This could be interpreted as a consistent clinical assessment by the observer and absence of observation bias.

Applying the high soy diet for two days should increase the protein content in the intestines and therefore affect the intestinal microbial flora. *B hyodysenteriae* should proliferate and be excreted in larger amounts resulting in a higher detection rate by PCR. A correlation between the presence and severity of diarrhoea and the *B hyodysenteriae* amounts excreted in the faeces had previously been confirmed (Magistrali and others 2014). In the present study, these effects could not be confirmed. The detection rate varied from 0.5 per cent to 1.5 per cent, which is low compared with other studies with detection rates of 10.5 per cent to 23.8 per cent (Barcellos and others 2000, Carvajal and others 2006). The herds in the present study had a previous diagnosis of *B hyodysenteriae* infection and, based on this, a higher detection rate was expected. Several reasons might explain the actual finding. *B hyodysenteriae* is discontinuously excreted as shown in a study where the bacterium could not be detected in a known positive herd (Heinonen and others 2000). In the present study, three herds tested *B hyodysenteriae* negative throughout the whole study period. A further reason might be that the sensitivity of the used TaqMan-PCR protocol, developed for clinically infected pigs, was too low to detect *B hyodysenteriae* in rectal swab material from subclinically infected pigs. These subclinically infected pigs might not have shed the bacterium at detectable levels (Harris and others 1999). Some other authors describe PCR assays as being less sensitive, when extracting *Brachyspira* DNA directly from faeces (Jacobson and others 2004a), or due to PCR inhibitors in faeces or extensive loss of DNA with the extraction kit (Corona-Barrera and others 2004). In order to establish an improvement in the detection rate, a culture for *B hyodysenteriae* with consecutive PCR as in previous studies (Råsbäck and others 2006, Song and Hampson 2009, Wilberts and others 2015) might have been necessary. However, one study showed only marginal differences in sensitivity of culture and PCR (Hartnack and others 2014). Whether the test sensitivity of culture combined with PCR or direct PCR from faeces in *B hyodysenteriae* subclinically infected pigs in Switzerland is comparable warrants further investigation.

Another reason for the low detection rate might be that feeding soy for two days is insufficient to increase the excretion of *B hyodysenteriae*. However, in the preliminary study the most significant changes in the faecal parameters were detected after applying soy for two days, and, thus, a two-day treatment period was selected. Furthermore, applying soy for only two days is more convenient at farm level than seven days, as performed in infection trials (Jacobson and others 2004b). Shedding of *B hyodysenteriae* two or three days after experimental infection may be influenced by the high infective doses as well as by repeated inoculation over two or three days (Jacobson and others 2004b). Bacterial counts in subclinically infected pigs of this study may be lower, which would require more time for sufficient propagation and thus for reaching detectable levels of the bacterium.

In conclusion, the present study showed that a high soy diet applied over two days influenced the faecal consistency and FDMC in growers, finishers and sows under field conditions. Further investigations with more sensitive diagnostic methods are needed to prove a potential influence of a high soy diet on the detection rate of *B hyodysenteriae* in subclinically infected herds.
